# Understanding GPs’ views and experiences of using clinical prediction rules in the management of respiratory infections: a qualitative study

**DOI:** 10.3399/BJGPO.2021.0096

**Published:** 2021-07-14

**Authors:** Hilda O Hounkpatin, Catherine Woods, Mark Lown, Beth Stuart, Geraldine M Leydon

**Affiliations:** 1 Primary Care Research Centre, School of Primary Care, Population Sciences and Medical Education, Faculty of Medicine, University of Southampton, Southampton, UK

**Keywords:** qualitative research, diagnosis, respiratory illness, general practice

## Abstract

**Background:**

Respiratory tract infections (RTIs) account for 60% of antibiotic prescribing in primary care. Several clinical prediction rules (CPRs) have been developed to help reduce unnecessary prescribing for RTIs, but there is a lack of studies exploring whether or how these CPRs are being used in UK general practice.

**Aim:**

To explore UK GPs’ views and experiences with regards to RTI CPRs, and to identify barriers and facilitators to their use in practice.

**Design & setting:**

A qualitative analysis of interviews with in-hours GPs working in the South and South West of England.

**Method:**

Semi-structured qualitative telephone interviews were conducted, digitally recorded, transcribed verbatim, and analysed using an inductive thematic approach. Patient and public involvement representatives contributed to study design and interpretation of findings.

**Results:**

Thirty-two GPs were interviewed. Some CPRs were more commonly used than others. Participants used CPRs to facilitate patient—clinician discussion, confirm and support their decision, and document the consultation. GPs also highlighted concerns including lack of time, inability of CPRs to incorporate patient complexity, a shift in focus from the patient during consultations, and limited use in remote consultation (during the COVID-19 pandemic).

**Conclusion:**

This study highlights the need for user-friendly CPRs that are readily integrated into computer systems, and easily embedded into routine practice to complement clinical decision-making. Existing CPRs need to be validated for other populations where demographics and clinical characteristics may differ, as well different settings including remote consultations and self-assessment.

## How this fits in

Several CPRs have been developed to predict outcomes of RTIs and help clinicians identify patients who may or may not benefit from antibiotics. There is a lack of UK qualitative studies exploring whether and how these CPRs are being used in practice. This study illustrates that some existing CPRs are not commonly used in practice and highlights concerns regarding their use including lack of time, awareness, and limitations to their use, both before and during the COVID-19 pandemic. The study highlights the need for CPRs that are user-friendly, easily embedded into practice, and validated for different populations and settings such as remote consultations and self-assessment.

## Introduction

Antimicrobial resistance remains a global public health problem.^1^ Reducing unnecessary and inappropriate use of antibiotics remains crucial to tackling antimicrobial resistance, particularly in primary care where most antibiotics are prescribed.^2,3^ Acute respiratory tract infections (RTIs) account for approximately 60% of antibiotic prescribing in UK primary care.^4,5^ Prescribing for RTIs is common, despite studies showing antibiotics have no or only marginal clinical benefit, and may even have side effects.^4^ Although UK prescribing rates have recently declined, more prudent prescribing is needed to reduce antimicrobial resistance.^2^

Clinical prediction rules (CPRs) or risk scores are tools that quantify the relevant contribution of patient characteristics — such as medical history, clinical examination, and diagnostic tests — to determine whether a patient is likely to have serious illness or complications, and can be used to assist management decisions.^6,7^ Such tools are particularly useful in reducing and managing clinical uncertainty.^6,7^ An increasing number of CPRs, for example STARWAVe to help predict hospitalisation in children, CURB-65 to predict pneumonia, and the Centor and FeverPAIN scores for acute sore throat, have been developed to help clinical teams determine who may benefit from an antibiotic prescription and reduce unnecessary consumption.^8,9^

Randomised controlled trials have shown CPRs are effective at reducing antibiotic prescription rates for RTIs. The National Institute for Health and Care Excellence (NICE) guideline NG84 recommends the Centor and FeverPAIN scores for managing sore throat.^9^ However, little is known about whether or how these CPRs are being used in practice.^10–14^ There is a lack of studies that have explored GPs’ use of CPRs in RTIs in UK primary care. One study used a survey to explore 401 UK GPs’ uptake of CPRs in a range of diseases and reasons for not using them, reporting poor use and awareness of CPRs.^15^ However, the study focused only on specified CPRs, and did not provide an in-depth exploration into GPs’ decision-making processes. A more comprehensive understanding of issues related to adoption of CPRs is needed to help inform further studies on the development, implementation, and evaluation of more effective CPRs and strategies to optimise their use in practice.

The current study aimed to explore UK GPs’ views and experiences with regards to RTI CPRs. It explored whether and how they are being used in practice, as well as facilitators and barriers related to the adoption of CPRs.

## Method

### Participant and recruitment

Local Clinical Research Networks (LCRNs; Wessex and West of England) were used to identify potential participants for interview. LCRNs advertised the study and provided a list of potential participants’ contact details.

Participants were purposively sampled to allow diverse characteristics in terms of age, years of experience, sex, and size and location of practice. In order to take part, the GPs had to be currently working in a general practice surgery with patients with RTIs. Snowball sampling was also used to improve recruitment towards the end of the study, whereby participants were asked to inform colleagues about the research. Eligible GPs were emailed a participant information sheet and consent form, and invited to take part in the telephone interview. Participants received a £50 Amazon voucher for their participation in the research study.

### Data collection

Semi-structured telephone interviews were conducted by HH, a non-clinical researcher who received training in qualitative interviews from the University of Southampton. Interviews were chosen as an optimal approach to gather relevant data on the individual experiences of GPs. Telephone interviews enabled recruitment from a broader geographical area and were convenient for the busy clinicians taking part.^16^ A semi-structured topic guide was used, as this allows participants to contribute their own views and allows unanticipated topics to be explored. The topic guide was informed by key topics within the existing literature, and discussions with public contributors. Participants were asked whether they were aware of and used CPRs for RTIs, how they used CPRs, perceived facilitators and barriers to using CPRs in clinical practice, and how CPRs can be best implemented in practice. The full topic guide is available in the Supplementary material. The interviews lasted between 30 and 60 minutes.

### Data analysis

Interviews were audio-recorded, transcribed verbatim, and analysed using inductive thematic analysis.^17^ HH read the transcripts repeatedly to ensure familiarity and coded each interview in detail, generating a list of codes and a coding manual to aid transparency for the full team, and ensuring consistency of coding over time.^18^ Field notes supported the analysis. CW, an experienced qualitative researcher, read and coded 20% of transcripts, and reviewed and refined codes with HH. The list of codes and key themes and issues was discussed and reviewed with the full research team. A lay summary (see Supplementary File 2) of the study and findings was shared with the public contributors and their views were incorporated into the analysis. NVivo (version 12) was used to organise and manage the data analysis process. Data collection and analysis was iterative and continued until data saturation was achieved.^19^

## Results

### Participants

Thirty-two GPs were interviewed. The demographics of participants are presented in Table 1. Most were White British, and the median age of participants was 44 (range 32–60) years. The median years since qualifying as a GP was 14 years (range 1–32). Most participants worked in urban and medium-sized practices.

**Table 1.** Participant characteristics

**Characteristic**	***n* (%**)
**Sex**	
Male	16 (50)
Female	16 (50)
**Age, years**	
Range	32–60
Median	44
**Ethnic group**	
White	27 (84.4)
Asian	5 (15.6)
**Years in general practice**	
Range	1–32
Median	14
**Location**	
Rural	9 (28.1)
Urban	18 (56.3)
Mixed or market town	5 (15.6)
**Size of practice**	
Small	3 (9.4)
Medium	16 (50)
Large	13 (40.6)

Most participants were aware of CPRs for acute RTIs. The most named CPRs were the FeverPAIN and Centor criteria for sore throat and the CRB-65 for pneumonia. More general CPRs, such as NEWS (National Early Warning Scorce) and EWS (Early Warning Score), were less commonly mentioned. Some participants reported that they hardly ever used CPRs, others used the CPRs occasionally, and a few almost always used CPRs to support their decision-making. Findings on participants’ views and experiences of using CPRs for RTIs are presented in four themes: 1) advantages of using CPRs; 2) concerns about their use; 3) the use of CPRs during the COVID-19 pandemic; and 4) implementation of CPRs in future patient consultations (Table 2). Illustrative quotations from participants are presented in the text and the participant number, sex, and age are presented in parentheses.

**Table 2.** GP reported advantages, concerns regarding CPRs for respiratory tract infections, their use in remote consultations, and implementation

**Themes**	**Subthemes**
1) Advantages of CPRs	**Useful for providing evidence for prescribing decisions to patients**
	**Evidence-based decision aid for clinicians**
	**Documenting what happened in the consultation**
2) Concerns about use of CPRs	**Patient complexity**
	**An intrusion on the consultation**
	**Time constraint as main barrier**
	**Issues with accuracy and interpretation**
3) Use of CPRs during COVID-19	**CPRs need to be adapted for remote consultations**
	**CPRs need to be validated for remote consultations**
	**Patients could complete CPR**
4) Implementation of CPS	**Integration into computer systems**
	**GP awareness**
	**CPRs need to be evidenced and endorsed**
	**Ideally validated in primary care population**
	**CPR design and length**
	**Changes to CPR to improve patient understanding**
	**Monetary incentives not needed**

CPRs = clinical prediction rules.

### 1) Advantages of using CPRs

#### Useful for providing evidence for prescribing decisions to patients

Most participants reported that the key benefit of using CPRs for acute RTIs was to provide evidence to justify prescribing decisions to patients:

*'I would, then, also use the score to validate so they can see in black and white that they don't fit the criteria at this stage.'* (GP21, male, 40)

*'Using a tool and saying,"Look, I've just measured these four things and I've given you a score and the guidelines are, and the guidance, and this is based on research", it’s quite useful in that way.'* (GP7, female, 58)

*'You can say there’s only like a ten per cent chance of this being a bacterial infection, and I think that’s useful rather than just saying, this is most likely to be.'* (GP32, male, 43)

Participants discussed how most patients felt reassured by them using CPRs in this way:

*'It’s almost like a mini second opinion that people sometimes want.'* (GP19, male, 40)

*'It is really useful to say “this is not just me doing this on a limb, we've used some specific things that are validated.” '* (GP3, female, 32)

Some participants also highlighted the importance of using CPRs to communicate risk to patients, support shared decision-making regarding treatment, and also manage expectations for antibiotics in the future:

*'*[It’s] *something else to input into the discussion with a patient and to give them an idea of how likely they are to benefit from antibiotics.'* (GP26, male, 60)

*'Then with that information, you can then, with the patient, look at that together, and then make a decision about whether antibiotics are going to be useful in this situation or not.'* (GP18, male, 52)

*'Going through those things can be very helpful in a patient’s understanding and also, it helps educate them for the next time they have a sore throat.'* (GP10, male, 43)

However, a few participants felt that patients were not always convinced by the evidence and that a thorough clinical examination was more important to help patients settle their concerns:

*'I think sometimes patients feel a bit like I'm a human being. I'm not just a set of numbers.'* (GP28, female, 38)

#### Evidence-based decision aid for clinicians

Many participants appreciated that CPRs were particularly helpful for reducing uncertainty as to whether a patient might benefit from antibiotics or not and helped with safety netting:

*'I guess there are some people where you're on the fence with whether you give them antibiotics. For those people, I suppose I have used it and thought, okay, well, the Centor criteria said this is what I should be doing, therefore I'm going to give it to them.'* (GP23, male, 38)

*'The ones that I'm like, really not sure, and then to objectively guide me as to whether I should do a delayed script maybe, or immediate antibiotics, that’s helpful.'* (GP8, female, 48)

Some participants reported using CPRs mainly to confirm or support their own clinical decision, while other participants reported using CPRs to guide their risk assessment:

*'If you're thinking this child, I think they're fine, my experience tells me they’re fine, and the score reflects that it’s quite good to sort of ‘belt and braces’ your clinical decision.'* (GP15, female, 47)

*'I've normally made a clinical decision anyway in my mind, but it’s nice to use it as a tool at the end to make a decision, a final decision.'* (GP5, female, 40)

*'It gives you a structure, making sure that you remember to ask the right questions.'* (GP22, female, 50)

Many participants felt that using CPRs was good practice and could help standardise care:

*'It can reinforce our opinion as being the same as our peers and this is understood wisdom.'* (GP7, female, 58)

*'It gives standardised messages to patients if everybody is using the same scores all the time.'* (GP21, male, 42)

Some participants felt that CPRs did not add much value to their decision-making:

*'If it’s not going to help you manage a patient, then I don't necessarily see the logic in completing it, for completing its sake.'* (GP14, female, 47)

*'It tells you what you already know, I feel.'* (GP29, male, 43)

#### Documenting what happened in the consultation

Some participants used CPRs to document the consultation and their decision. Those who used CPRs in this way reported doing so mostly for medical-legal reasons but also to allow colleagues to refer to their consultation:

*'Having something documented regarding a tool that you've used stands you, probably, in a better stead.'* (GP12, female, 36)

*'It’s helpful to have it there. So if someone was looking over your notes they could think, “okay, well, you've made a decision and I can follow your thinking more clearly.” '* (GP25, male, 34)

Participants with greater years since qualifying reported less experienced GPs may find CPRs more useful to build confidence not to prescribe. Participants from small practices generally felt more confident applying CPRs as they had a good knowledge of their patients. However, all participants highlighted that CPRs could only be used as an adjunct to clinical judgement, and ultimately clinical acumen would override a CPR score.

### 2) Concerns about using CPRs

#### Patient complexity

All participants reported that CPRs could not consider patient complexity and were less helpful for certain patients including those patients with recurrent infections, existing comorbidities, or psychosocial factors:

*'I think Centor’s more helpful in school-age children and younger adults rather than the elderly* [older patients]*, because I think, the elderly, it’s more how systemically unwell they are* *.'* (GP4, male, 53)

*'I think you can't fit people into boxes and I think score systems are brilliant for looking at very limited things, but they don't look at the whole person.'* (GP22, female, 50)

*'I think with the more complicated people with multiple conditions and chronic disease, then they are a bit less useful.'* (GP2, female, 35)

#### An intrusion on the consultation

Some participants described how using CPRs negatively affected the consultation:

*'You're trying to work out and focus on the patient. Sometimes, going away and doing a score gets yourself quite focused on the computer.'* (GP27, male, 60)

*'I guess the skill would be trying to weave it into just the smooth history and examination without making it feel like you were doing that chunky tick-box thing.'(*GP31, male, 38)

#### Time constraint as a main barrier

Many participants emphasised that lack of time was the main barrier to using CPRs:

*'They’re kind of the same things you’re asking them anyway, so I think it just sometimes feels a bit tedious.'* (GP20, female, 40)

*'Unless you’ve got them in your head, they’re just not happening. It’s too bloody busy.'* (GP27, male, 60)

Some participants felt overburdened by the number of existing CPRs and protocols, as well as changes overtime:

*'It’s just one more thing we’re asked to use.'* (GP1, female, 60)

*'Every area that we cover has its own scoring system, so if I tried to memorise them, that would be fantastic, but six months later there would be a whole bunch of new ones.'* (GP29, male, 43)

#### Issues with accuracy and interpretation

A few participants described challenges of the subjective nature of the questions in Centor and FeverPAIN, and how this could lead to inaccurate assessments:

*'Often, what patients tell you and what is a reality are two very different things.'* (GP30, male, 36)

*'With more subjective stuff it can sometimes be really hard to tie people down with feelings.'* (GP11, female, 59)

Generally, participants with more years of experience seemed to have more reservations about using CPRs. They discussed how CPRs were not widely available at the time of their training and that they had always relied on their clinical judgment. Some also described being less comfortable with information technology. Participants who felt they were low prescribers also found CPRs to be less useful.

### 3) Use of CPRs during the COVID-19 pandemic

#### CPRs need to be adapted for remote consultations

Owing to COVID-19, many consultations were being conducted remotely. There were mixed feelings regarding the use of CPRs in remote consultations. Some felt that they could not confidently apply these CPRs without a physical examination, while others reported they were still able to complete these CPRs using video consultations, high-quality photos, and patient-reported measures:

*'I'd probably be bending the rules slightly, there, if I couldn't do all of the bits of it, but it might give me a bit of an indication.'* (GP31, male, 38)

*'The scores are only as reliable as the examination that you're doing at the time.'* (GP5, female, 40)

#### CPRs need to be validated for remote consultations

When asked, almost all participants described the importance of validating CPRs for remote consultation:

*'If they* [CPRs] *were validated scores based on remote assessment, that would be really useful because then you'd feel that it was more geared to the actual situation that you're in* *.'* (GP19, male, 40)

*'It would be useful if they* [CPRs] *were history based or simple examination based that you could do over a video phone* *.'* (GP7, female, 58)

#### Patients could complete CPR

Many participants felt CPRs could be used as part of pre-assessment telephone or online triage systems. Some participants discussed the use of images or instruction sheets to help patients take reliable measurements. Similar views were expressed from participants from urban and rural practices, and medium and large practices.

### 4) Implementation of CPRs

#### Integration into computer systems

There was strong agreement among participants regarding factors that would increase GP uptake of CPRs. All participants described the need for CPRs to be easily accessible and well-integrated into their computer systems:

*'Most of these things don't take very long; it’s just knowing where they are.'* (GP9, male, 46)

*'They're helpful, but then they're only helpful if they're there.'* (GP13, female, 48)

*'Part of it is that you've got to remember them.'* (GP7, female, 58)

#### GP awareness

Many participants also discussed the importance of increasing GP awareness of CPRs, through constant reminders, discussions with colleagues, and promotion at regional or national level:

*'The stuff that’s being encouraged by CCGs* [clinical commissioning groups] *and publicised, I think is very easy to find, but some of the other scores are you'd have to go looking for them to find them* *.'* (GP12, female, 36)

#### CPRs need to be evidenced and endorsed

Participants spoke of how they were more likely to use a CPR if there was a strong evidence base for it and if it was developed by experts and endorsed by guidelines:

*'I feel confident if it’s a local or NICE guideline; I have full confidence in that.'* (GP24, female,44)

*'The ones that are promoted in GP updates or The BMJ, or end up in clinical guidelines, I'm tending to believe that they have been properly validated and tested, and therefore are useful and I should be using them.'* (GP26, male, 60)

#### Ideally validated in primary care population

Many participants also discussed how CPRs should be developed and validated in primary care populations:

*'You're dealing with different populations, so a test that’s, say, is quite good at picking up that somebody’s got sepsis in hospital is going to be vastly, over-predict sepsis in a community population.'* (GP4, male, 53)

*'They've* [CPRs] *got to be developed for the target population and the target use, so for general practice* *.'* (GP15, female, 47)

#### CPR design and length

All participants reported that simple, user-friendly CPRs were most useful and easily embedded into their routines:

*'It’s* [Centor’s] *easy. It’s not got too many factors. I don't need a template to remind me of all the questions and I don't need a template to add it up* *.'* (GP13, female, 48)

*'The Centor—it comes up populated within the template. It’s very easy to just click the buttons, to fill it in. Also, it’s a short temple.'* (GP24, female, 44)

#### Changes to CPR to improve patient understanding

Some participants recognised the need for CPRs to include additional information and materials to further facilitate patient–clinician communication and help patients better understand the use of antibiotics:

*'A link to a page which would say* — *which would tell you, or you can look up your score and it would give some sort of visual aid to understanding what that meant. I think that would be useful* *.'* (GP6, male, 41)

#### Monetary incentives not needed

Most participants felt monetary incentives were not needed to increase uptake of CPRs as they described a great responsibility to reduce antibiotic resistance as a sufficient driver for their use:

*'There is a huge incentive which is to stop antibiotic resistance, and I think we feel as UK GPs that that’s a huge bonus of us, or responsibility.'* (GP10, male, 43)

## Discussion

### Summary

This study explored GPs’ views and experiences of using CPRs in the management of acute RTIs. Participants generally expressed favourable opinions on CPRs and recognised advantages of using them alongside clinical judgment. Some CPRs were more commonly used than others. Participants used CPRs in different ways: to evidence their decision-making to patients, and to support, confirm, or document their prescribing decisions. Important barriers to their use were highlighted, such as lack of time, the inability of CPRs to account for patient complexity, limitations of the subjective elements of the CPRs, a shift in focus from the patient during the consultation, and limited use in remote consultation. Participants suggested simple, user-friendly CPRs that are well-integrated into computer systems and endorsed by guidelines are needed to increase awareness, accessibility, and usability. Furthermore, participants expressed a need for existing CPRs to be validated for different populations and remote consultations.

### Strengths and limitations

A strength of this study was the inclusion of participants from a mix of practices (urban versus rural, medium versus large) in different geographical areas, and with a broad range in years of experience, which allowed a range of views to be captured. The semi-structured qualitative approach allowed an in-depth understanding of emerging concepts. A limitation was that some participants had been or were involved in work or research on antibiotic prescribing, and this may have influenced their views on CPRs. It is likely that participants who took part in the study had different (and possibly more favourable) view of CPRs than those who did not. Participants were predominantly White British, which meant views from ethnic minority GPs were not explored extensively. Similarly, there were fewer participants from small practices.

Often in the management of acute respiratory infections, the clinician will first make a diagnosis based on the history or examination (or both), and then use different strategies to refine this diagnosis and rule out competing possibilities.^20^ For example, a clinician may hypothesise that the patient has a viral infection and then will use a CPR to estimate the probability of a bacterial infection. This in turn will influence the treatment strategy, which may vary by country or context (that is, delayed prescribing for sore throat in the UK versus further near-patient diagnostic testing in the US).^21^ However, CPRs are only one diagnostic reasoning strategy that might be used. Clinicians may use other strategies, for example, comparing symptoms and signs with previous patterns of cases to reach a diagnosis. GPs differ in their use of diagnostic strategies and the strategy used may also depend on the condition or presenting symptoms.

### Comparison with existing literature

This is the first UK qualitative interview study to explore views and experiences of CPRs for RTIs, overall, and in the context of remote consultations. The findings are consistent with the wider literature on CPRs, which has found that key influences of uptake of CPRs are time, integration into computer systems, and lack of relevance to some patients.^22–25^ Similarly, evidence-based medicine and patient–clinician communication have been reported as the main reasons for use of CPRs more generally.^22^ The findings are also in line with a theory that suggests barriers, such as GP attitude (for example, lack of familiarity or motivation to use CPRs), and patient and environmental factors (for example, clinical guidelines or practice factors) may play a role in the use of CPRs.^25,26^ Some participants reported a lack of perceived value of CPRs or preference for clinical judgment, particularly among more experienced participants, which has been described in previous studies.^15,22,27^ The finding that CPRs for RTIs may often be used to provide evidence to justify prescribing decisions to patients, or to confirm their own recommendation rather than aid decision-making, is consistent with findings on CPRs for other conditions.^28^ In contrast to a survey study published in 2014 that found 76% of surveyed GPs reported that they had never heard of the Centor score,^15^ the present study found that most participants were now aware of the score. This suggests an improvement in awareness of some existing scores, for example, for sore throat. The present study extends the current literature on use of CPRs by highlighting the need for CPRs that are useful and validated for remote consulting.

### Implications for research and practice

This study suggests that many existing CPRs for RTIs are not routinely used in practice. Existing CPRs may be useful for remote consultations and pre-assessment triage systems, but further work is needed to explore how they can be safely adapted and validated. Any adapted CPRs will need to be user-friendly, auto-populated, and easily embedded into routine practice. Endorsement in trusted guidelines and promotion at CCG and practice level, as well as integration into computer systems, may help increase awareness and normalise their use.
